# Obesity Is Associated with an Increase in Pharmaceutical Expenses among University Employees

**DOI:** 10.1155/2015/298698

**Published:** 2015-02-08

**Authors:** Julie A. Gazmararian, David Frisvold, Kun Zhang, Jeffrey P. Koplan

**Affiliations:** ^1^Department of Epidemiology, Rollins School of Public Health, Emory University, Atlanta, GA 30322, USA; ^2^Department of Economics, University of Iowa, Iowa City, IA 52242, USA; ^3^Department of Health Policy and Management, Rollins School of Public Health, Emory University, Atlanta, GA 30322, USA; ^4^Emory Global Health Institute, Emory University, Atlanta, GA 30322, USA

## Abstract

*Objective*. To examine costs associated with obesity in an employee population and factors associated with increased costs. *Methods*. We used data from the Physical Activity and Life Style (PALS) study, a randomized prospective design evaluating three interventions to increase physical activity among physically inactive nonfaculty university employees (*n* = 454). The primary exposure variable, obesity (measured by body mass index), was obtained from the in-person baseline survey. Covariates were obtained from the baseline survey and included demographic characteristics and health status. Data from the baseline survey was linked with administrative data to determine pharmaceutical, inpatient, outpatient, and total health care costs for three years. Average monthly expenditures for obese and nonobese individuals were compared using *t*-tests and a two-part multivariate regression model adjusted for demographic and socioeconomic characteristics and health behaviors. *Results*. Although in-patient and outpatient expenses were not associated with obesity, pharmaceutical expenditures were $408 or 87.2% higher per year ($468 versus $876) for obese individuals than for nonobese individuals, which reflected poorer health behaviors and health status of obese adults. *Conclusion*. Awareness of the costs associated with obesity among employees can stimulate employers to make the investment in providing employer-sponsored wellness and health improvement programs to address obesity.

## 1. Introduction

In the United States, obesity among adults has increased markedly since 1980 [1]. In 2011-2012, 34.9% of adults 20–74 years old were obese [2]. A higher body weight is associated with an increased incidence of a number of health conditions, including diabetes mellitus, cardiovascular disease, and nonalcoholic fatty liver disease, and with an increased risk of disability [3]. Obesity is associated with a modestly increased risk of all-cause mortality [4, 5].

The social and economic costs of obesity are high [2]. Overweight and obesity attributable medical spending/expenditures accounted for 9.1% of total annual US medical expenditures or $78.5 billion in 1998 [6]. Increases in the proportion of and spending on obese people relative to people of normal weight account for 27% of the rise in inflation-adjusted per capita health care spending between 1987 and 2001. Increases in obesity prevalence alone account for 12% of the growth in health spending [7]. Other analyses consistently document that obese individuals have a significantly higher use of health care services (including outpatient and inpatient visits) and associated costs (such as pharmacy costs) than nonobese patients [3, 6–19].

Not only does obesity place a significant burden on our health care system, it also has an impact on the costs to employers due to lost productivity, absences, underperformance, and higher insurance premiums. Data from the Medical Expenditures Panel Survey (MEPS) for 2000–2004 showed that absenteeism costs associated with obesity total $4.3 billion annually in the USA [17]. Among a sample of municipal workers, after accounting for age, gender, race, smoking behavior, and educational attainment, BMI predicted both average annual health care costs and work absence hours [18]. Using data from 61 US employers' health plan members' claims experiences between January 2000 and December 2004 showed that diagnosed, nondrug medical expenses attributable to obesity accounted for 21.3% of lifestyle health risks (accidents/injuries, alcohol/substance use, high cholesterol, high blood pressure, obesity, poor prenatal care, lack of exercise, smoking, stress, and poor dental hygiene) and 2.8% of all medical costs for those aged 19 to 64 years. Interestingly, this data also indicated that up to age 64 years (particularly those aged 55 to 64 years), females' obesity costs markedly exceed males [19]. Among manufacturing employees, moderately or extremely obese workers (BMI ≥ 35) experienced a 4.2% health-related loss in productivity, 1.2% more than all other employees, which equates to an additional $506 annually in lost productivity per worker [20].

Finally, a 2007 study explored the relationship between BMI and number and types of workers' compensation claims, associated costs, and lost workdays among 11,728 health care and university employees. Results indicated a clear linear relationship between BMI and rate of claims. Employees in obesity class III (BMI ≥ 40) had 11.65 claims per 1,000 full time employees (FTEs), while recommended-weight employees had 5.80: lost workdays 183.73 per 100 FTEs for overweight versus 14.19 for recommended-weight employees; medical claims costs ($51,091 versus $7,503 per 100 FTEs), and indemnity claims costs ($59,178 versus $5,396 per 100 FTEs). The claims most strongly affected by BMI were related to lower extremity, wrist or hand, and back; pain or inflammation, sprain or strain, and contusion or bruise [21].

These data strongly indicate that employers should explore workplace interventions to reduce these costs [22]. Reducing obesity (along with tobacco use and inactivity) should be a priority for employers seeking to lower the incidence and severity of chronic illness and the associated demand for health services. Awareness of the costs associated with obesity among employees can stimulate employers to make the investment in providing employer-sponsored wellness and health improvement programs to address obesity [23]. The primary objective of this analysis is to examine the costs associated with obesity in an employee population and factors associated with increased costs.

## 2. Materials and Methods

The Physical Activity and Life Style (PALS) study was conducted at Emory University in Atlanta, Georgia, that employs over 12,000 staff and faculty [24]. The PALS study used a randomized prospective design to evaluate three interventions to increase physical activity among Emory employees. The analysis described in this paper utilized data (e.g., height and weight) from the baseline survey conducted for the main PALS study and linked this information with administrative data to determine health care costs.

### 2.1. Study Population

The PALS study population includes departments with at least six nonexempt (i.e., clock in and out for work each day) employees. Departments were excluded if the majority were employed by Emory University's Hospital system or if the primary location for the department was not on the university's main campus.

Individual employees were* excluded* for the following factors that precluded the need for an intervention, interfered with receiving the intervention, or interfered with conducting in-person interviews: (1) reported meeting CDC guidelines for physical activity, as assessed with a brief exercise screener; (2) worked nights; (3) worked off campus; (4) expected to be absent from work for more than a month in the next year (e.g., maternity leave, sabbatical); or (5) worked less than 20 hours/week. Faculty members and employees defined as* exempt* (i.e., not clocking in and out for work) were excluded because of their flexible work schedule.

Additional details about how departments and individuals were contacted to participate in PALS are provided elsewhere [24]. If determined eligible and willing, PALS participants were scheduled to have five data collection points over 9 months. This study used data from the baseline surveys conducted between April 2006 and March 2007: (1) Baseline Part A (demographics, health attitudes and behaviors, and work environment), on-line or on paper, and (2) Baseline Part B (physical activity recall, height and weight, and health literacy), in-person. The study protocol received approval from the Emory Institutional Review board.

### 2.2. Measures

The primary exposure variable was obesity (BMI ≥ 30). Height and weight measures were taken of participants by trained interviewers during the in-person baseline and nine-month follow-up surveys. The interviewers followed standard protocol used by the National Health and Nutrition Examination Survey for measuring height and weight [25]. Other covariates in this analysis were obtained from the baseline survey and included gender, race, age, income, education, marital status, job classification, health literacy skills, health-related behaviors (smoking status, gym membership, and attendance), self-reported health status, and physical and mental health (the number of unhealthy days in the past 30 days and the number of chronic conditions).

Claims data for all medical and pharmaceutical expenses were provided by Aetna, Blue Cross Blue Shield (BCBS), Medco, Towers Perrin, and United Behavior Science (UBH) for the period spanning May 2005 to February 2008 (34-month period). The overwhelming majority (74%) of employees were enrolled in the Aetna Point of Service plan, and plan choice did not vary by weight status (BMI) of study participants. Furthermore, all employees had the same prescription drug coverage plan. We created measures of average monthly costs for the following medical costs: total medical expenses, pharmaceutical expenses, inpatient expenses, and noninpatient expenses including physician visit, outpatient, emergency room, and other expenses. Pharmaceutical expenses are measured based on claims from Medco and Towers Perrin. Inpatient and noninpatient expenses are measured based on claims from Aetna, BCBS, and UBH. Total medical expenses then are obtained by aggregating pharmaceutical, inpatient, and noninpatient expenses. We also create measures of inpatient and noninpatient expenses for conditions related to obesity. We define obesity-related conditions using the claims-included primary ICD-9 codes related to visits for obesity (V65.3, V65.41, V69.0, V69.1, V77.8, V85.3, 278.00, 278.01, and 278.8), or related to diabetes, hypertension, coronary heart disease, and hyperlipidemia. We also created measures of average monthly out-of-pocket costs that are defined as the aggregate of copayments, coinsurance, and deductibles during this period for all medical expenses. Finally, we created average monthly sick leave hours obtained from the Emory University human resources records for the period spanning May 2005 to February 2008 (34-month period).

### 2.3. Analysis

We calculated the characteristics of the study population separately for nonobese (BMI < 30), obese (BMI ≥ 30), and morbidly obese (BMI ≥ 35) individuals. We also calculated the mean monthly medical expenses for each category of expenditures (total, pharmaceutical, inpatient, noninpatient, obesity-related inpatient, and obesity-related noninpatient expenses) for each weight category, the percent of individuals with positive expenses during any month of the 34-month period, and the mean monthly expenses for individuals with positive expenses. We initially examined whether the expenses for nonobese individuals are equivalent to those for obese individuals and whether the expenses for nonobese individuals are equivalent to those for morbidly obese individuals using *t*-tests. A *P* value of 0.05 was used to determine statistical significance.

We also examined the relationship between medical expenses and obesity, conditional on individual demographics and health-related behaviors, using multivariate regression analysis. We estimated the probability that an individual has positive medical expenses for each category of expenditures using a logit model. We calculated average partial effects and heteroskedasticity-robust standard errors. Finally, we estimate a two-part regression model to account for the nonpositive expenditures and the right skewed distribution of medical expenditures, where the first part consists of a logit model predicting the probability of positive expenditures and the second part consists of a generalized linear model with a log link and a gamma distribution for individuals with positive expenditures. Bootstrapped standard errors are calculated with 400 replications. Two sets of covariates are used for all regressions. The first set of covariates includes sex, race (white, black, and other), marital status (married, single, and other), and age. The second set of covariates includes health behaviors and chronic conditions as defined above to examine whether the relationship between obesity and medical expenses is mediated by poor health behaviors and chronic conditions. Additional covariates denoting the treatment group that the individual was assigned to in the PALS study had no impact on the estimates of the relationship between obesity and medical expenses and thus are excluded from the results described below.

The average monthly sick leave hours used is positive for almost all individuals in the sample. Thus, we examined the relationship between obesity and average monthly sick leave hours using ordinary least squares regression models. We control for the same two sets of covariates as used in the models of medical expenditures.

## 3. Results

Unless otherwise noted, all results mentioned are statistically significant at *P* < 0.05.  Table 1 displays the differences in demographics and health behaviors of nonobese, obese, and morbidly obese individuals. In this sample, obese individuals are more likely to be black, are less likely to have completed college or graduate school, are less likely to attend a gym at least once a week, are more likely to report their health as fair or poor, have more unhealthy days in the past month, and have more chronic conditions than nonobese individuals. Morbidly obese individuals are more likely to be female, more likely to be black, are less likely to have completed college or graduate school, are less likely to attend a gym at least once a week, are more likely to report their health as fair or poor, have more unhealthy days in the past month, and have more chronic conditions than nonobese individuals. All other variables included in this analysis were not significantly different between obese or morbidly obese and nonobese individuals.

Table 2 demonstrates that the monthly average of all medical expenditures is $75 more per month ($905 annually) for obese individuals than nonobese individuals and is $83 more per month ($992 annually) for morbidly obese individuals than nonobese individuals. These differences are not due to differences in whether these groups of individuals have any expenditures, since nearly all individuals have out-of-pocket expenses at least once during this 34-month period. Of the total medical expenditures, the monthly average of out-of-pocket expenditures is only $12 more per month ($138 annually) for obese individuals than nonobese individuals and is only $17 more per month ($209 annually) for morbidly obese individuals than nonobese individuals.

The largest differences in types of expenditures are differences in pharmaceutical expenses, with obese individuals spending $34 and morbidly obese individuals spending $43 more per month than nonobese individuals. Again, the differences in pharmaceutical expenses are not due to differences in the percent of each group having an expense. Although the average inpatient and noninpatient expenses of obese individuals are higher than nonobese individuals, these differences are not statistically significant. The average monthly inpatient expenses for all causes and causes related to obesity are small in magnitude; thus, we do not focus on these categories in the remaining tables. The average noninpatient expenses from causes related to obesity are higher among obese and morbidly individuals, compared to nonobese individuals, which reflects the differences in incurring this type of expense.

The mean differences in Table 2 could reflect the demographic differences between obese and nonobese individuals shown in Table 1.  Table 3 displays the results of the two-part models that condition on demographics and models that also condition on socioeconomic characteristics, health behaviors, and chronic conditions. As shown in column (1), total medical expenses for obese individuals are $81.7 higher per month than nonobese individuals. As stated above, this result is driven by pharmaceutical expenses; obese individuals have pharmaceutical expenses that are $38.6 higher per month than nonobese individuals, as shown in column (3). Additionally, obese related noninpatient expenses are $12.8 higher per month, as shown in column (7), and total out-of-pocket expenses are $13.4 higher per month, as shown in column (9) for obese individuals than nonobese individuals. However, as shown in the even columns, socioeconomic characteristics, health behaviors, and chronic conditions mediate the relationship between obesity and medical expenses. As shown in column (4), controlling for these behaviors reduces the estimate for total medical expenditures to $52.2 and it is no longer statistically significant. Further analyses suggest that obese individuals are more likely to have chronic conditions, which largely explains the decrease in the estimate from column (1) to column (2). Controlling for these variables also substantially decreases the estimates for pharmaceutical expenses and total out-of-pocket expenses. There are no statistically significant differences in inpatient or noninpatient expenditures.

Estimates of the relationship between obesity and average monthly sick leave hours taken are shown in Table 4. As shown in column (1), obese individuals use 1.44 more hours of sick leave per month than nonobese individuals. Controlling for socioeconomic status, health behaviors, and health status reduces the estimate to 1.03 more hours of sick leave per month, which remains statistically significant.

## 4. Discussion

Overall, our results in a population of university employees indicated that total medical expenditures were higher for obese individuals than nonobese individuals, which reflect the poorer health behaviors and health status of obese adults. The differences in total medical expenditures were primarily due to higher pharmaceutical expenses, as being obese is not predictive of higher inpatient or outpatient expenses. Further, being obese increased the probability of having noninpatient expenses for obesity related medical conditions but did not increase the amount of noninpatient expenses for these conditions. Additionally, obese individuals had higher out-of-pocket expenses, but these expenses were small in magnitude and also reflected the poorer health behaviors and health status of obese adults.

This study has at least five strengths. First, cost data were available for three years. This time period is longer than most other studies that have examined costs using MEPS data or employer costs associated with obesity. Second, we had actual measurements of obesity, rather than most studies that have relied on self-reported height and weight data, which is important due to recent results that self-reported measures lead to underestimation of the relationship between obesity and medical costs [26]. Third, we had more recent cost data available compared to others that examined older data [27]. Fourth, we had additional information about study participants, including demographic characteristics and mental and physical health measures, which allowed us to examine whether the higher medical costs of obesity are due to obesity or the resulting comorbidities. Finally, results from this study had implications for worksite interventions by potentially motivating employers to provide programs for employees who are overweight/obese.

Despite the numerous strengths of this study, there are at least three limitations. First, the study population only included individuals covered by private health insurance plans. However, the majority of employed Americans are covered by private health plans. Therefore, this data is useful to specifically examine employer costs associated with obesity. Second, data were only available from employees who were covered by Emory health insurance plans. Employees who were covered by other plans (e.g., spouse coverage) were not included; however, 90.5% of Emory employees are covered by Emory health plans. Finally, the sample size is relatively small, although fairly similar to MEPS data.

Our results suggest that the additional total medical expenditures for obese employees are $905 per year and for morbidly obese employees are $992 per year. These differences are mostly driven by pharmaceutical expenses and are attributable to demographics, socioeconomic characteristics, health-related behaviors, and health status. In contrast, Finkelstein et al. find that the increase in medical spending due to obesity is $1140 per year (2008 dollars) for privately insured individuals and that inpatient and noninpatient costs account for approximately three-fourths of the additional expenses [6]. Further, Cawley et al. find that obesity increases medical expenses by $4393 per year (2005 dollars) for privately insured adults [17].

A difference between our results and previous results in the literature is that we are able to control for a wide array of individual characteristics and we find that the additional medical expenses reflect the health status of obese individuals as opposed to obesity causing an increase in medical expenditures. A second difference is that we focus on a sample of employees offered generous health insurance plans as a benefit of employment. Our results show that, provided with generous health insurance plans, the additional out-of-pocket expenditures by obese individuals are minimal. In contrast, the premiums for these health insurance plans have increased in recent years. For example, the full premium for the most generous insurance plan increased by 61% between 2004 and 2008 for family coverage, and employees paid only 1% of this increase. During this period, the deductible for services provided within the core network remained at $0. The high additional medical expenditures attributable to obesity shown in the previous literature are not reflected in out-of-pocket expenditures for these employees because most expenses are covered under the available insurance plans and the additional costs due to obesity are incorporated into the health insurance premiums. Thus, the additional costs of obesity are only partially paid by obese employees through higher premiums and these health insurance plans redistribute costs from obese to nonobese employees who pay the same insurance premiums.

## Figures and Tables

**Table 1:** Characteristics of study population (means or % {standard errors}) by obesity status.

	Nonobese (BMI < 30)	Obese (BMI ≥ 30)	*P* value (nonobese = obese)	Morbidly obese (BMI ≥ 35)	*P* value (morbidly obese = nonobese)
Number of observations	255	199		96	
Female	60.8% (0.031)	62.8% (0.034)	0.659	74.0% (0.045)	0.022
Race					
White	42.7% (0.031)	36.7% (0.034)	0.191	31.3% (0.047)	0.050
Black	45.5% (0.031)	59.8% (0.035)	0.003	65.6% (0.048)	0.001
Other	11.8% (0.020)	4.0% (0.014)	0.003	3.1% (0.018)	0.013
Age	41.3 (0.7)	43.4 (0.7)	0.057	43.2 (1.0)	0.166
Income	$49,235 (1694.7)	$48,982 (1823.0)	0.919	$48,437 (2581.5)	0.802
Educational status					
High school	16.1% (0.023)	15.6% (0.026)	0.885	13.4% (0.035)	0.534
Some college	32.9% (0.029)	50.8% (0.035)	0.000	56.7% (0.050)	0.000
College	33.7% (0.030)	23.1% (0.030)	0.014	20.6% (0.041)	0.017
Masters	16.1% (0.023)	8.0% (0.019)	0.010	6.2% (0.024)	0.015
Marital status					
Married	47.1% (0.031)	47.7% (0.035)	0.885	44.8% (0.051)	0.704
Single, never married	31.4% (0.029)	27.1% (0.032)	0.326	32.3% (0.048)	0.869
Other	21.6% (0.026)	25.1% (0.031)	0.373	22.9% (0.043)	0.786
Job classification					
Facility management	22.7% (0.026)	25.1% (0.031)	0.404	27.8% (0.045)	0.319
Non-FM	77.3% (0.026)	73.9% (0.031)	0.404	72.2% (0.047)	0.319
Adequate or above Health literacy skills	50.6% (0.031)	54.3% (0.035)	0.436	53.1% (0.051)	0.672
Current smoker	13.3% (0.021)	8.5% (0.020)	0.109	12.5% (0.034)	0.834
Gym membership	22.4% (0.026)	23.1% (0.030)	0.847	21.9% (0.042)	0.924
Gym attendance once a week or more	18.8% (0.024)	10.1% (0.022)	0.009	6.3% (0.025)	0.004
Fair or poor health Status	14.5% (0.022)	31.7% (0.033)	0.000	40.6% (0.050)	0.000
Unhealthy days^a^	4.9 (0.38)	6.3 (0.595)	0.037	7.7 (0.980)	0.001
Number of chronic conditions	0.47 (0.050)	0.86 (0.067)	0.000	0.96 (0.104)	0.000

^a^Unhealthy days is measured as the number of days during the past 30 days when either physical or mental health was not good.

**Table 2:** Health care expenses of study population (means or % {standard errors}) by obesity status.

	Nonobese (BMI < 30)	Obese (BMI ≥ 30)	*P* value (obese = nonobese)	Morbidly obese (BMI ≥ 35)	*P* value (morbidly obese = nonobese)
Number of observations	255	199		96	

Total medical expenses					
Monthly average costs	180.1 (17.8)	255.5 (28.4)	0.020	262.8 (35.8)	0.024
Percent of individuals with total costs >0	86.7% (0.021)	86.4% (0.024)	0.942	89.6% (0.031)	0.463
Monthly average costs if costs >0	208.7 (20.0)	308.1 (32.8)	0.007	296.7 (38.9)	0.030
Monthly average Total out-of-pocket medical costs	31.3 (2.80)	42.8 (4.34)	0.021	48.7 (7.11)	0.006

Pharmaceutical expenses					
Monthly average costs	39.0 (7.24)	73.0 (12.4)	0.013	82.3 (18.9)	0.009
Percent of individuals with total costs >0	73.3% (0.028)	77.4% (0.030)	0.323	80.2% (0.041)	0.185
Monthly average costs if costs >0	58.4 (10.6)	100.9 (16.5)	0.026	105.3 (23.6)	0.037

Inpatient expenses					
Monthly average costs	19.2 (7.63)	29.1 (7.70)	0.370	22.3 (7.21)	0.815
Percent of individuals with total costs >0	7.8% (0.017)	16.1% (0.026)	0.006	16.7% (0.038)	0.015
Monthly average costs if costs >0	272.3 (91.0)	214.3 (42.5)	0.525	164.7 (32.9)	0.341

Noninpatient expenses					
Monthly average costs	121.9 (11.8)	153.3 (18.1)	0.132	158.2 (22.5)	0.127
Percent of individuals with total costs >0	83.5% (0.023)	80.9% (0.028)	0.468	85.4% (0.036)	0.668
Monthly average costs if costs >0	145.9 (13.5)	193.1 (21.8)	0.055	185.2 (25.2)	0.145

Obese related inpatient expenses					
Monthly average costs	6.31 (6.22)	6.32 (3.06)	0.999	2.39 (2.17)	0.702
Percent of individuals with total costs >0	1.18% (0.007)	5.53% (0.016)	0.008	5.21% (0.023)	0.024
Monthly average costs if costs >0	536.7 (524.4)	114.3 (46.1)	0.128	45.9 (40.6)	0.252

Obese related noninpatient expenses					
Monthly average costs	8.20 (2.32)	20.9 (4.50)	0.008	25.3 (8.16)	0.007
Percent of individuals with total costs >0	29.0% (0.028)	49.2% (0.036)	0.000	54.2% (0.051)	0.000
Monthly average costs if costs >0	29.8 (7.94)	42.8 (8.72)	0.291	46.6 (14.5)	0.280

**Table 3:** The relationship between obesity and average monthly medical expenses (two-part model estimates) (*N* = 454).

	Total medical expenses		Pharmaceutical expenses		Noninpatient expenses		Obese related noninpatient expenses		Total out-of-pocket medical expenses	
	(1)	(2)	(3)	(4)	(5)	(6)	(7)	(8)	(9)	(10)
Obese	81.7^**^	52.2	38.6^**^	16.3	32.3	15.6	12.8^**^	12.7^**^	13.44^**^	8.09
	(36.3)	(35.1)	(16.7)	(13.8)	(21.9)	(21.7)	(5.3)	(5.8)	(6.11)	(5.73)

Notes: heteroskedasticity-robust standard errors in parentheses. Two-part model estimates are estimated using a logit model in the first part and a generalized linear model with a log link and a gamma distribution for the second part. Covariates in the odd-numbered columns include gender, race, marital status, and age. Covariates in the even-numbered columns also include socioeconomic characteristics (education, income, and occupation category), health behaviors (smoking status, gym membership and attendance, health literacy, and health status), and the number of chronic conditions.

^*^ *P* < 0.1, ^**^P < 0.05, and ^***^P < 0.01.

**Table 4:** The relationship between obesity and average monthly sick leave hours (*N* = 424).

	Monthly sick leave hours	
Models	(1)	(2)
Obese	1.44^***^	1.03^**^
	(0.38)	(0.44)

Notes: heteroskedasticity-robust standard errors in parentheses. The covariates included but not shown are the same as the covariates included in Table 3.

^**^P < 0.05, and ^***^P < 0.01.
